# Disentangling the relationship between glucose, insulin and brain health: A UK Biobank study

**DOI:** 10.1111/dom.70353

**Published:** 2025-12-12

**Authors:** Andrew C. Mason, Nasri Fatih, Reecha Sofat, Christopher T. Rentsch, Liam Smeeth, Krishnan Bhaskaran, Nish Chaturvedi, Victoria Garfield

**Affiliations:** ^1^ Department of Pharmacology and Therapeutics University of Liverpool Liverpool UK; ^2^ Unit for Lifelong Health and Ageing, Institute of Cardiovascular Science University College London London UK; ^3^ Nuffield Department of Population Health, Big Data Institute University of Oxford Oxford UK; ^4^ Department of Non‐Communicable Disease Epidemiology London School of Hygiene and Tropical Medicine London UK

**Keywords:** dementia, epidemiology, genetics, glycaemia, Mendelian randomisation

## Abstract

**Background:**

Glycaemic traits are associated with poorer brain health and dementia risk. Recent advances in genetic instruments for specific glycaemic markers enable an in‐depth investigation of the likely nature of associations and underlying mechanisms between diabetes‐related mechanisms and brain health and dementia.

**Methods:**

We used two‐sample Mendelian randomisation (MR) in the UK Biobank (UKB) (maximum *N* = 357 883 White British, mean age 56.9 years, 54% female) applying inverse‐variance weighted MR as our main estimator alongside MR‐Egger, weighted median estimator (WME) and Mendelian Randomization Pleiotropy RESidual Sum and Outlier (MR‐PRESSO) as sensitivity tests. Instruments were 53 insulin resistance, 109 fasting glucose, 48 fasting insulin and 15 2‐h post‐load glucose genetic variants with variant–outcome effects estimated adjusting for 10 PCs. We checked core MR assumptions and sought to replicate results in an independent Alzheimer's dementia genome‐wide association study (GWAS).

**Results:**

In UKB, higher 2‐h post‐load glucose was associated with a 69% increased Alzheimer's dementia risk (odds ratio 1.69 [95% confidence interval 1.38–2.07]), though this did not replicate in an independent GWAS. Fasting insulin, fasting glucose and postprandial glucose did not influence total brain, hippocampal or white‐matter hyperintensity volumes.

**Discussion:**

The association between elevated 2‐h post‐load glucose and increased Alzheimer's risk supports a potential role for postprandial hyperglycaemia in dementia. The lack of associations between fasting or postprandial glucose and hippocampal, total‐brain or white matter hyperintensity volumes suggests this risk may operate independently of gross structural atrophy.

**Conclusion:**

Genetically proxied postprandial hyperglycaemia contributes to increased Alzheimer's risk in mid‐life, warranting replication in other populations and ancestries to confirm and clarify underlying mechanisms.

## 1 BACKGROUND

Epidemiological studies have long suggested that hyperglycaemia, diagnosed type 2 diabetes mellitus (T2DM) and insulin resistance (IR) strongly relate to worse brain health, specifically increasing the risk of cognitive decline and dementias.^1^, ^2^, ^3^, ^4^, ^5^, ^6^ The mechanisms behind these relationships are poorly understood^7^ and we have yet to make substantial progress in the treatment of individuals who present with both diabetes and cognitive dysfunction.^8^ The nature and direction of these relationships also remain largely elusive, with some evidence pointing towards a bidirectional association,^7^ which makes prevention and intervention even more difficult, as this suggests a vicious cycle in which diabetes could lead to dementia, with the dementia itself then causing additional diabetes complications.^9^

Mendelian randomisation (MR) is a genetic epidemiology method which helps overcome some of the core limitations of observational studies, namely residual confounding and reverse causation.^10^ To date, MR studies have largely investigated Alzheimer's dementia (AD) as an outcome, finding no causal relationship from T2DM to AD.^11^, ^12^, ^13^ Pathways to cognitive decline and dementia involve a combination of vascular and neurocognitive mechanisms that may act either independently or in concert.^14^, ^15^ Diabetes is more strongly related to the vascular pathways, but there is evidence that it also has neurotoxic consequences.^16^ A combination of vascular and neurocognitive mechanisms which either act independently or in synchrony is likely to lead to cognitive decline and, ultimately, dementia.^17^, ^18^ In a previous MR study, we showed that HbA_1c_ and diabetes are unlikely to be the direct causal culprits underlying poorer cognitive function, brain health and both all‐cause dementia and AD.^11^ However, T2DM consists of distinct sub‐phenotypes characterised by different grades of insulin responsiveness,^19^ and HbA_1c_ has inconsistent associations with fasting and post‐load glucose.^19^

Now, with the availability of better genetic instruments for diabetes‐related mechanisms such as IR, fasting glucose (FPG), fasting insulin (FI) and 2‐h post‐load glucose (2hPG),^20^ the present study aimed to investigate whether (i) IR, FPG, FI and 2hPG are causally related to specific neuroimaging brain health outcomes and (ii) IR, FPG, FI and 2hPG are causally associated with the risk of all‐cause dementia, AD and/or vascular dementia (VD).

## 2 METHODS

### 2.1 Study design

Two‐sample MR (which exploits genome‐wide association summary statistics derived in non‐overlapping samples) was used to mitigate biased results due to the ‘winners’ curse (the over‐estimation of genetic associations which are common in one‐sample MR).^21^ An important advantage of using two‐sample MR is that it allows sensitivity analyses to identify unbalanced (directional) horizontal pleiotropy (described under *Statistical analyses*), which is required to satisfy MR assumptions.

### 2.2 Sample

Full details of the UK Biobank (UKB) cohort are published elsewhere.^22^ Briefly, UKB consists of 500 000 males and females from the general UK population, aged 40–69 years at baseline (2006–2010). Due to the low number of participants with AD diagnoses of other ancestry, the sample was restricted to those of White British ancestry. There was a maximum of 357 883 participants of white British ancestry with both genotype and all the outcomes and covariates of interest for our study (Table 1).

**TABLE 1:** Sample characteristics for UKB participants considered in our study (*n* = 357 883).

Sample characteristics	
	Mean (SD)/*N* (%)/median (IQR)
Age (years)	56.9 (8.0)
Sex (male)	187 272 (46)
Socioeconomic deprivation score	−1.55 (3.81)
Smoking	
*Ever*	162 011 (45)
Physical activity (number of days)	2.4 (3.18)
Hippocampal volume (mm^3^)	3993 (408)
White matter hyperintensity volume (mm^3^)^a^	3056 (4667)
Body mass index (kg/m^2^)	27.4 (4.8)
Systolic blood pressure (mmHg)	140.2 (19.7)
Triglycerides (mmol/mol)^a^	1.75 (1.02)
Total cholesterol (mmol/mol)	4.65 (0.95)
C‐reactive protein (mg/L)^a^	2.6 (4.4)
Stroke	9218 (2.6)

^a^ Median (IQR).

Abbreviations: UKB, UK Biobank.

Ethical approval for this work was granted by the Northwest Haydock Research Ethics Committee of the Health Research Authority from the United Kingdom. All necessary patient/participant consent has been obtained, the appropriate institutional forms have been archived, and any patient/participant/sample identifiers included are masked to those (e.g., hospital staff, patients or participants themselves) outside the research group so cannot be used to identify individuals.

### 2.3 Genotyping and quality control (QC) in UKB

A number of 487 409 UKB participants were genotyped using 1 of 2 customised genome‐wide arrays that were imputed to a combination of the UK10K, 1000 Genomes Phase 3 and the Haplotype Reference Consortium reference panels, which resulted in 93 095 623 autosomal variants.^23^ We then applied additional variant‐level quality control (QC) and excluded genetic variants with: Fisher's exact test <0.3, minor allele frequency <1% and a missing call rate of ≥5%. Individual‐level QC meant that we excluded participants with excessive or minimal heterozygosity, more than 10 putative third‐degree relatives as per the kinship matrix, no consent to extract DNA, mismatches between self‐reported sex and genetic sex, missing QC information and non‐European ancestry (based on how individuals had self‐reported their ancestry and the similarity with their genetic ancestry, as per a principal component analysis of their genotype).

### 2.4 Outcomes: structural brain magnetic resonance imaging and dementia

Structural brain magnetic resonance imaging scans were performed by UKB in a subsample of participants using standard protocols, as described in detail elsewhere.^24^ The post‐processed measures derived by UKB and used in this study included: mean hippocampal volume (cm^3^)—adjusted for head size, total brain volume (cm^3^)—and volume of white matter hyperintensities (WMH, mm^3^). WMH volume was log‐transformed due to positive skew. The maximum number of participants with WMH volume with neuroimaging outcomes available for this study was 46 788, after exclusion of *n* = 224 individuals who were outliers (+3SD from the mean) and did not pass genetic QC. We report results in cm^3^ for hippocampal and total brain volume and exponentiated betas/percentages for WMH volume.

UKB provided algorithmically defined all‐cause dementia, AD and VD, captured using International Classification of Diseases (ICD) ‐10 codes in linked Hospital Episode Statistics data, as well as from death registry data and primary care. Coded diagnoses were compared with clinical expert adjudication of full‐text medical records. Details of ICD‐10 and primary care Read codes are presented in Tables S1 and S2. More in‐depth information on the algorithm by Wilkinson et al. can be found elsewhere.^25^ We used the most up‐to‐date number of dementia cases (maximum *N* = 8896) in UKB, who also had complete data for genotype and other phenotypes (e.g., covariates).

### 2.5 Statistical analyses

Associations between genetic variants and outcomes of interest were computed using methods in PLINK version 2.0. MR analyses were performed using the *TwoSampleMR* R package^26^ and methods contained therein.

#### 2.5.1 Selection of genetic variants for exposures: IR, FPG, FI and 2‐h glucose

For IR, we selected the same 53 genetic variants used by Dale et al.,^27^ which were part of a 2017 MAGIC Consortium genome‐wide association study (GWAS) and included 188 577 individuals,^20^ which had no overlap with the UKB samples. For the remaining glycaemic exposures (FPG/FI/2hPG), we selected genetic variants from the European summary statistics from the latest and GWAS published by the MAGIC Consortium. Details are published elsewhere^20^, ^28^ but briefly, this GWAS consisted of up to 281 416 individuals. We selected independent single‐nucleotide polymorphisms (SNPs) for inclusion in our analyses at *r* ^2^ < 0.01 within a 250‐kb region. We used the clump function in Plink 2.0 with these parameters, which yielded 109, 48 and 15 SNPs for FPG, FI and 2‐h glucose, respectively. We aligned alleles from the MAGIC GWAS with our UKB data prior to analysis, by multiplying any misaligned variants' beta coefficient by −1. As we did not have directly observed data for these exposures in UKB, we were unable to estimate the variance explained (*R* ^2^) and *F*‐statistic in our sample. We approximated the *F*‐statistic by calculating them in the original sample from the MAGIC GWAS, as previously recommended.^29^ We used the ‘*t‐*statistic’ formula which yielded *F*‐values of 89, 49, 107 and 54 for IR, FI, FPG and 2hPG, respectively.

#### 2.5.2 Main MR analyses

We firstly performed linear/logistic regression to examine the associations between our genetic instruments for all 3 exposures and our outcomes in PLINK 2.0, adjusted for 10 genetic principal components to account for residual population stratification. Subsequently, inverse‐variance weighted (IVW) MR was implemented as our main model. The IVW calculates the effect of a given exposure (e.g., FPG) on an outcome of interest (e.g., hippocampal volume) by taking an average of the genetic variants' ratio of variant‐outcome (*SNP → Y*) to variant‐exposure (*SNP → X*) relationship estimated using the same principles as a fixed‐effects meta‐analysis.^30^ We also performed standard MR sensitivity analyses, including MR‐Egger regression (which provides an intercept term to indicate the presence or absence of unbalanced horizontal pleiotropy)^30^, ^31^ and the weighted median estimator (WME—which may yield robust estimates when up to 50% of the genetic variants are invalid). For additional robustness, we also used Mendelian Randomization Pleiotropy RESidual Sum and Outlier (MR‐PRESSO) as another test of horizontal pleiotropy. Results are presented as exponentiated *ß* coefficients (multiplicative effect size) for WMHV, all‐cause dementia/AD/VD risk, unit differences in hippocampal volume (cm^3^) and total brain volume (cm^3^).

#### 2.5.3 MR assumption checks

MR has three strict assumptions that must be met for study results to be valid:

- The association between the genetic variants for the exposure and the exposure itself must be strong and robust (this means that these associations have usually been replicated and validated via GWAS). *This assumption was met because our genetic variants were all from large‐scale recently published GWAS*.
- The association between the genetic variants (for the exposure) and the outcome must only be via the exposure under study, otherwise this is known as unbalanced horizontal pleiotropy and may bias MR results. *This assumption was assessed using the sensitivity analyses mentioned above* (*i.e., MR‐Egger, WME and MR‐PRESSO*).
- The genetic variant–outcome relationship is unconfounded. One way to test this is associations between the genetic variants (for the exposure) and common covariates of the relationship under study (e.g., the exposure SNPs should not be associated with factors such as smoking). *We assessed this assumption by regressing multiple covariates (body‐mass index, socioeconomic deprivation, systolic blood pressure, total cholesterol, triglycerides, C‐reactive protein and physical activity—for which outliers >3 standard deviations were removed—smoking and stroke) on the IR, FPG, FI and 2hPG SNPs. We applied a Benjamini‐Hochberg false discovery rate (BH‐FDR) of 0.05 to account for multiple testing*.

#### 2.5.4 MR replication analyses: AD

Replication analyses were carried out using summary statistics from the latest GWAS of AD,^28^ which had a maximum of 111 326 AD cases (UKB‐specific cases: AD‐by‐proxy, *n* = 46 828 and diagnosed cases, *n* = 2447) and 677 663 controls. AD‐by‐proxy cases were defined as participants who reported a family history of dementia. Given the inclusion of these UKB AD cases, the sample overlap between our study and Bellenguez et al. was ~2%. We extracted log(betas) and standard errors for 14 2hPG, 107 FPG, 48 FI and 53 IR SNPs, respectively.

## 3 RESULTS

The overall sample consisted of 357 883 participants. The participants had a mean age of 56.9 years and were more likely to be female (54.1%). Twenty‐seven percent of participants reported ever smoking, 20% belonged to the most deprived group and the overall mean BMI was 26.7 kg/m^2^. More details on the participants' characteristics are shown in Table 1.

### 3.1 Associations between FI, FPG and 2hPG, IR and dementia

**FIGURE 1:**
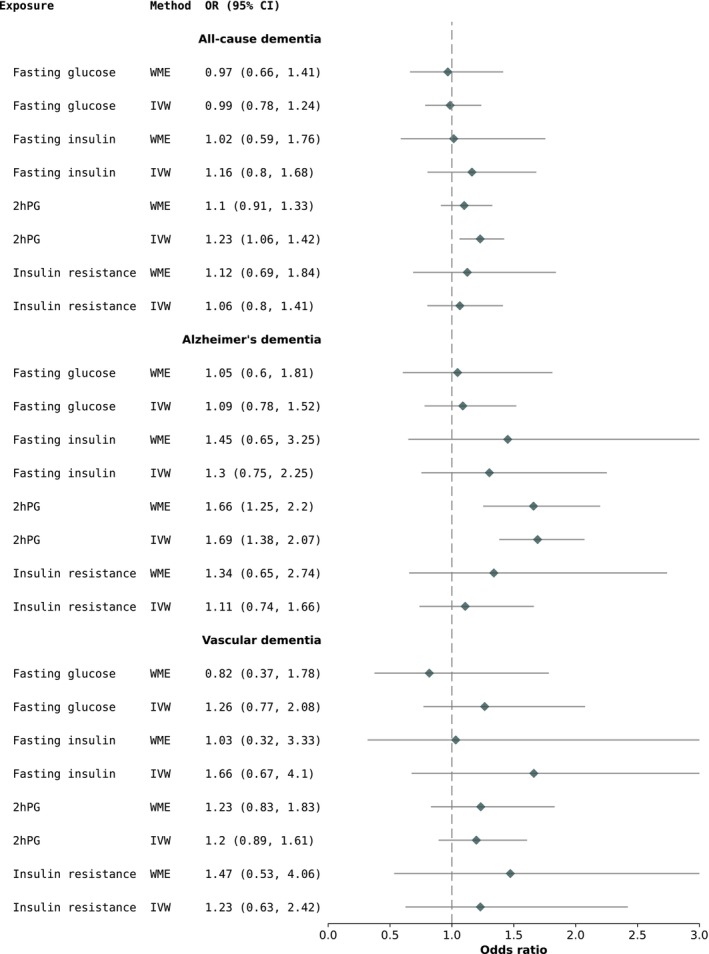
MR results for the relationship between glycaemic exposures and all‐cause dementia (*N* = 8896), Alzheimer's dementia (*n* = 4028) and vascular dementia (*n* = 1925) in UKB. 2hPG: 2‐h post‐glucose; IVW, inverse‐variance weighted; MR, Mendelian randomisation; OR, odds ratio; UKB, UK Biobank; WME, weighted median estimate.

Using MR, we found no evidence of associations between FI/FPG/IR and all‐cause dementia or AD (Figure 1). Using IVW, there was an association between 2hPG and a 23% increased risk of all‐cause dementia (Figure 1). Additionally, elevated 2hPG was associated with a 69% increased risk of AD under the IVW model, which was consistent, albeit somewhat smaller when using a WME (Figure 1). We also observed no issues with unbalanced horizontal pleiotropy, as all the MR‐Egger intercept *p*‐values were >0.05 and MR‐PRESSO detected no outlier SNPs for any instrument, reporting global test *p*‐values >0.05.

### 3.2 Associations between FI, FPG, 2hPG, IR and neuroimaging outcomes

**FIGURE 2:**
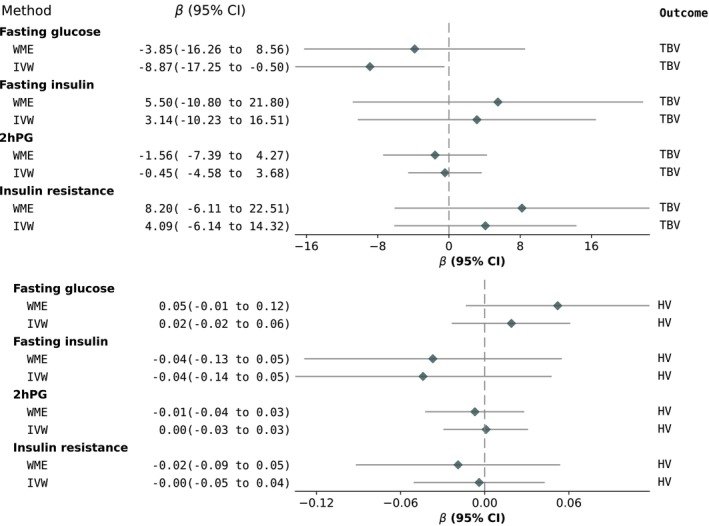
MR results for the relationship between glycaemic exposures and hippocampal and total brain volume (*n* = 46 828). 2hPG, 2‐h post‐glucose; HV, hippocampal volume; IVW, inverse‐variance weighted; MR, Mendelian randomisation; TBV, total brain volume; WME, weighted median estimate.

**FIGURE 3:**
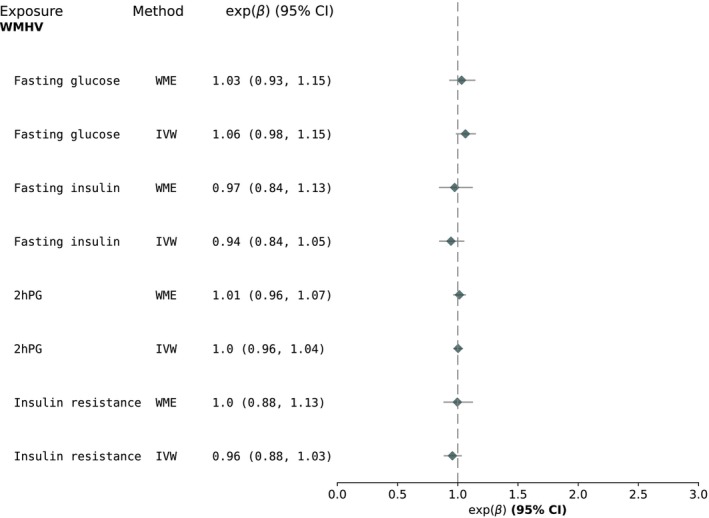
MR results for the relationship between glycaemic exposures and white matter hyperintensity volume (*n* = 46 828). 2hPG, 2‐h post‐glucose; IVW, inverse‐variance weighted; MR, Mendelian randomisation; WME, weighted median estimate; WMHV, white matter hyperintensity volumes.

We found no associations between FPG/2hPG/IR/FI and hippocampal volume, total brain volume or white matter hyperintensity volume (Figures 2 and 3). All MR‐Egger *p*‐values were indicative of no unbalanced horizontal pleiotropy (*p* > 0.05). The MR‐PRESSO global test indicated no horizontal pleiotropy for all associations, except for IR → total brain volume (*p* = 0.003). Two outlier SNPs were detected, but this did not change the result as it remained towards the null (Table S9).

### 3.3 Results from MR assumption check III: Associations between genetic instruments and covariates

We checked for associations between the 2hPG/FI/FPG/IR SNPs and a range of common covariates. We found associations between some 2hPG/FI/FPG/IR SNPs and CRP, triglycerides, smoking status, SBP, body mass index, total cholesterol, physical activity and deprivation at a BH‐FDR 0.05 threshold (Table S6).

### 3.4 Replication analysis: Glycaemic traits and AD

**FIGURE 4:**
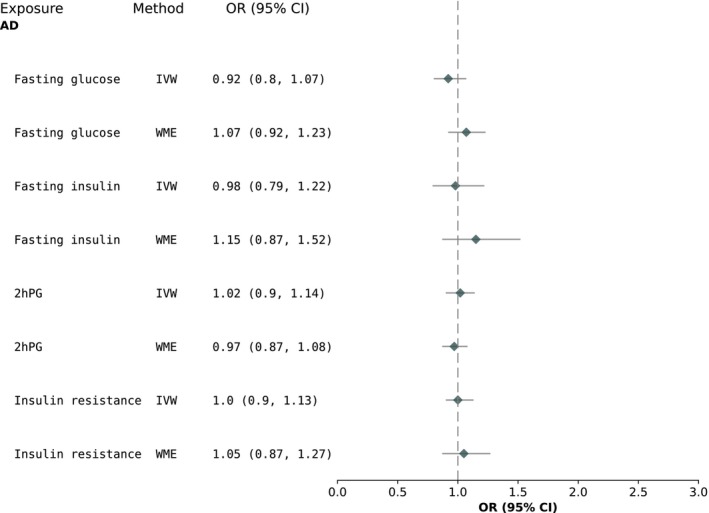
MR replication results for the association between glycaemic exposures and AD (max *n*_cases_ = 111 326). 2hPG, 2‐h post‐glucose; AD, Alzheimer's dementia; IR: Insulin resistance; IVW, inverse‐variance weighted; WME, weighted median estimate.

In our replication analyses using the summary statistics from the latest GWAS of AD, we found no associations between our glycaemic exposures and AD (Figure 4).

## 4 DISCUSSION

### 4.1 Overview of findings

In this two‐sample MR study, we found evidence of an association between 2hPG and a 69% increased risk of AD in the UKB. However, this association did not replicate using a different GWAS dataset from Bellenguez et al.^28^ We found no evidence of associations between our glycaemic exposures and all‐cause dementia or VD. Our MR analyses of FPG, 2hPG, FI and IR with volumetric brain outcomes showed no relationship with TBV, WMHV or hippocampal volume.

### 4.2 Findings in context

We previously published an MR study in the UKB and found no relationship between T2DM or HbA1c levels and measures of cognitive function and brain health.^32^ In the current analyses, however, we considered additional markers of diabetes‐related mechanisms and found a relationship between 2hPG and AD risk. Although this was found both using the IVW and WME approaches, reducing the likelihood that the results are due to bias from invalid instruments or pleiotropy, there remains the possibility that this finding is due to chance.

Previous observational and MR studies have suggested that 2hPG is a glycaemic trait that strongly predicts poorer cardiovascular outcomes.^33^, ^34^ Our findings suggest that the genetic predisposition for this marker of postprandial glucose is also associated with an increased risk for AD. There were some indications that the IR and FPG models also followed a similar trend to the 2hPG (i.e., higher risk of AD particularly for the WME models) albeit the confidence intervals of the odds ratios overlapped with 1, and thus, this finding should be interpreted with caution.

How postprandial hyperglycaemia may be related to AD is poorly understood. In this study, we failed to observe evidence of a relationship between 2hPG and both hippocampal and total brain volume. This perhaps suggests that the underlying mechanism of this association may not occur through the characteristic global and/or hippocampal atrophy of AD. Future MR studies should examine whether genetically instrumented 2hPG is associated with the pathological markers of AD (i.e., tau and amyloid) to further shed the light on how the mechanisms of postprandial hyperglycaemia manifest in the brain. The failure to find an association between 2hPG and WMHV also suggests that this relationship may not manifest itself via vascular pathways; albeit, future studies should re‐examine these associations with more sensitive measures of cerebrovascular disease such as those relating to microvascular pathology. For example, evidence suggests that the shape and location of white matter lesions may be more important in type 2 diabetes.^35^

The replication analyses we carried out using summary statistics from the latest GWAS of AD did not show a similar 2hrPG–AD association. One explanation could be that the UKB analyses consist of a more general population sample while Bellenguez et al.'s^28^ cases/controls were specifically selected for dementia‐related traits. Once again, such discrepancy regarding how AD was ascertained in the two different datasets could introduce heterogeneity in the data, potentially diluting or altering the strength of the associations and our ability to replicate this finding. Bellenguez et al.^28^ combined multiple cohorts with varying diagnostic criteria, as well as AD proxy cases, which may have increased phenotypic heterogeneity.

The failure to find a convincing association between our glycaemic exposures and the volumetric measures is largely consistent with our previous MR analyses of the relationship between HbA1c/diabetes and brain and cognitive health.^32^ However, the associations for the WME, IVW and MR‐PRESSO were in the same directions (but with wider confidence intervals in the WME).

Our analyses showed that MR assumptions were largely met. While we did observe associations between some of the glycaemic exposures and covariates such as CRP, triglycerides and cholesterol, such associations may be suggestive of vertical pleiotropy, where SNPs affect these factors through glycaemia, maintaining the causal chain without introducing bias. Alternatively, these associations may highlight shared biological pathways connecting glycaemic regulation with metabolic or inflammatory processes. Future analyses should explore whether these SNPs are vertically pleiotropic by performing via MR mediation analyses. Importantly, MR‐PRESSO did not detect horizontal pleiotropy (except for IR‐TBV, but this result was null using IVW and WME, and remained null after removal of two outlier SNPs), as per the global test, which gives us confidence that these SNP‐covariate associations do not violate this MR assumption.

### 4.3 Strengths

One of the strengths of this study is that it used the UKB, one of the largest sources of imaging samples in the world. Our MR approach generated strong genetic instruments, as all *F*‐statistics were >10, indicating no weak instrument bias.^36^ Another strength is that we performed a replication analysis in an independent dataset. We also used a two‐sample MR approach employing GWAS summary statistics from non‐overlapping samples for all our glycaemic exposures and brain health outcomes. Using independent datasets gives us more confidence in our results by reducing biases such as the Winner's curse and weak instrument bias and increasing our statistical power.^21^ Non‐overlapping samples also reduce confounding, as each GWAS sample has independent trait distributions strengthening the directionality of associations by mitigating the risk of reverse causation.^21^

### 4.4 Limitations

The UKB is subject to a well‐documented ‘healthy volunteer’ bias, where participants tend to be healthier, better educated and more health‐conscious than the general population.^37^ Also, the UKB imaging subsample may represent a more selective group that may differ systematically from the broader cohort,^38^ potentially skewing associations with the imaging outcomes affecting the generalisability of MR findings. Additionally, we acknowledge that the statistical power for the imaging outcomes is lower in comparison to our analyses, which used the whole UKB cohort. This bias may have impacted our MR analyses by underestimating effects, particularly for traits or diseases influenced by lifestyle or socioeconomic factors. Furthermore, the restricted age range of UKB participants, primarily between 40 and 69 years at recruitment, may obscure genetic effects that manifest earlier or later in life, particularly for age‐related conditions such as dementia, restricting insights into life‐course trajectories of these traits. While genetic variants are fixed at conception and consequently reflect the life‐long exposure‐outcome association, it is possible that the phenotypic expression of an exposure (e.g., declining insulin sensitivity in older adults) may change with age, and this may have influenced our power to detect associations.

The potential misclassification of dementia outcomes in the UKB, which relies on hospital records, death registries and self‐reports, is also an important caveat. Misclassification may bias estimates and hinder the ability to distinguish between dementia subtypes, such as AD versus VD, reducing the reliability of findings. Despite the UKB's overall large sample size, the limited number of cases for certain outcomes, such as certain dementia subtypes (e.g., frontotemporal dementia), meant that we could not analyse these conditions. This would increase the risk of false negatives and restrict the ability to perform meaningful subgroup analyses. Together, these factors emphasise the importance of interpreting MR findings within the context of these constraints and validating results through external datasets when possible. We also only analysed White British participants due to low dementia case numbers in other ancestries. Future research should explore these associations in diverse populations to ensure generalisability across genetic backgrounds and disease risks. More research is also required to extrapolate our findings to clinical cohorts, such as those with metabolic diseases (e.g., severe type 2 diabetes).

## 5 CONCLUSIONS

In this UKB MR study which explored the genetic risk of a breadth of glycaemic markers and brain health outcomes, we observed an association between postprandial hyperglycaemia and higher AD risk. The former finding was specific to UKB and not in the chosen replication sample. Thus, future research is needed to confirm this and, if replicated, better understand the precise mechanisms through which this may occur. Future studies should also explore whether this association manifests in non‐European samples and aim to disentangle the mechanisms via which insulin may exert a positive effect on brain health.

## AUTHOR CONTRIBUTIONS

VG conceived the study idea. ACM performed the statistical analyses. ACM, VG and NF drafted the manuscript. NC, LS, CTR and KB provided important intellectual input and discussions related to the interpretation of the results. All individuals acknowledged have reviewed and approved their affiliation with this manuscript.

## FUNDING INFORMATION

This work is funded by the Professor David Matthews Non‐Clinical Fellowship to VG (ref: SCA/01/NCF/22). KB is funded by a Wellcome Senior Research Fellowship (220283/Z/20/Z). RS: NIHR Research Professor, NIHR303160 was funded by the NIHR for this research project. The views expressed in this publication are those of the author(s) and not necessarily those of the NIHR, NHS or the UK Department of Health and Social Care.

## CONFLICT OF INTEREST STATEMENT

NC serves on Data Monitoring and Safety Committees for trials sponsored by AstraZeneca.

## CONSENT TO PARTICIPATE

The authors confirm that all necessary patient/participant consent has been obtained, and the appropriate institutional forms have been archived, and that any patient/participant/sample identifiers included were not known to anyone (e.g., hospital staff, patients or participants themselves) outside the research group so they cannot be used to identify individuals.

## Supporting information


**Table S1.** Genetic instrument information for IR, 2hPG, FI and FPG.
**Table S2.** Associations between 2hrPG SNPs and confounders.
**Table S3.** Associations between FPG SNPs and confounders.
**Table S4.** Associations between FI SNPs and confounders.
**Table S5.** Associations between FI SNPs and confounders.
**Table S6.** Benjamini–Hochberg FDR‐corrected associations between SNPs and covariates.
**Table S7.** ICD‐10 codes for Alzheimer's disease (AD) in UK Biobank.
**Table S8.** Primary care Read codes (version 2) for Alzheimer's disease.
**Table S9.** MR‐PRESSO results.

## Data Availability

UK Biobank data were openly available before the initiation of the study, and it can be found by following this link: https://biobank.ndph.ox.ac.uk/showcase/search.cgi. This work is linked to UK Biobank approved project #422316.

## References

[1] Gudala K , Bansal D , Schifano F , Bhansali A . Diabetes mellitus and risk of dementia: a meta‐analysis of prospective observational studies. J Diabetes Investig. 2013;4:640‐650. 10.1111/jdi.12087 PMC4020261 24843720

[2] Cukierman T , Gerstein HC , Williamson JD . Cognitive decline and dementia in diabetes – systematic overview of prospective observational studies. Diabetologia. 2005;48:2460‐2469. doi:10.1007/s00125-005-0023-4 16283246

[3] McCrimmon RJ , Ryan CM , Frier BM . Diabetes and cognitive dysfunction. Lancet. 2012;379:2291‐2299. 22683129 10.1016/S0140-6736(12)60360-2

[4] Biessels GJ , Despa F . Cognitive decline and dementia in diabetes mellitus: mechanisms and clinical implications. Nat Rev Endocrinol. 2018;14:591‐604. doi:10.1038/s41574-018-0048-7 30022099 PMC6397437

[5] Ravona‐Springer R , Luo X , Schmeidler J , et al. Diabetes is associated with increased rate of cognitive decline in questionably demented elderly. Dement Geriatr Cogn Disord. 2010;29:68‐74. 20130405 10.1159/000265552 PMC2840245

[6] Xue M , Xu W , Ou YN , et al. Diabetes mellitus and risks of cognitive impairment and dementia: a systematic review and meta‐analysis of 144 prospective studies. Ageing Res Rev. 2019;55:100944. 31430566 10.1016/j.arr.2019.100944

[7] Biessels GJ , Nobili F , Teunissen CE , Simó R , Scheltens P . Understanding multifactorial brain changes in type 2 diabetes: a biomarker perspective. Lancet Neurol. 2020;19:699‐710. 32445622 10.1016/S1474-4422(20)30139-3

[8] Srikanth V , Sinclair AJ , Hill‐Briggs F , Moran C , Biessels GJ . Type 2 diabetes and cognitive dysfunction—towards effective management of both comorbidities. Lancet Diabetes Endocrinol. 2020;8:535‐545. 32445740 10.1016/S2213-8587(20)30118-2

[9] Ojo O , Brooke J . Evaluating the association between diabetes, cognitive decline and dementia. Int J Environ Res Public Health. 2015;12:8281‐8294. 26193295 10.3390/ijerph120708281 PMC4515722

[10] Davey Smith G , Ebrahim S . ‘Mendelian randomization’: can genetic epidemiology contribute to understanding environmental determinants of disease? Int J Epidemiol. 2003;32:1‐22. 12689998 10.1093/ije/dyg070

[11] Garfield V , Farmaki AE , Eastwood SV , et al. HbA1c and brain health across the entire glycaemic spectrum. Diabetes Obes Metab. 2021;23:1140‐1149. 33464682 10.1111/dom.14321 PMC8261644

[12] Hagenaars SP , Gale CR , Deary IJ , Harris SE . Cognitive ability and physical health: a Mendelian randomization study. Sci Rep. 2017;7:1‐7. 28572633 10.1038/s41598-017-02837-3 PMC5453939

[13] Østergaard SD et al. Associations between potentially modifiable risk factors and Alzheimer disease: a Mendelian randomization study. PLoS Med. 2015;12:1‐16. 10.1371/journal.pmed.1001841 PMC4469461 26079503

[14] Kisler K , Nelson AR , Montagne A , Zlokovic BV . Cerebral blood flow regulation and neurovascular dysfunction in Alzheimer disease. Nat Rev Neurosci. 2017;18:419‐434. 28515434 10.1038/nrn.2017.48 PMC5759779

[15] Nelson AR , Sweeney MD , Sagare AP , Zlokovic BV . Neurovascular dysfunction and neurodegeneration in dementia and Alzheimer's disease. Biochim Biophys Acta Mol Basis Dis. 2016;1862:887‐900. 10.1016/j.bbadis.2015.12.016 PMC4821735 26705676

[16] Fotuhi M , Do D , Jack C . Modifiable factors that alter the size of the hippocampus with ageing. Nat Rev Neurol. 2012;8:189‐202. 22410582 10.1038/nrneurol.2012.27

[17] Schneider JA , Arvanitakis Z , Bang W , Bennett DA . Mixed brain pathologies account for most dementia cases in community‐dwelling older persons. Neurology. 2007;69:2197‐2204. 17568013 10.1212/01.wnl.0000271090.28148.24

[18] Korf ESC , White LR , Scheltens P , Launer LJ . Brain aging in very old men with type 2 diabetes: the Honolulu‐Asia Aging Study. Diabetes Care. 2006;29:2268‐2274. 17003305 10.2337/dc06-0243

[19] Ahlqvist E , Storm P , Käräjämäki A , et al. Novel subgroups of adult‐onset diabetes and their association with outcomes: a data‐driven cluster analysis of six variables. Lancet Diabetes Endocrinol. 2018;6:361‐369. 29503172 10.1016/S2213-8587(18)30051-2

[20] Chen J , Spracklen CN , Marenne G , et al. The trans‐ancestral genomic architecture of glycemic traits. Nat Genet. 2021;53:840‐860. 34059833 10.1038/s41588-021-00852-9 PMC7610958

[21] Lawlor DA . Commentary: two‐sample Mendelian randomization: opportunities and challenges. Int J Epidemiol. 2016;45:908‐915. 27427429 10.1093/ije/dyw127 PMC5005949

[22] Sudlow C , Gallacher J , Allen N , et al. UK biobank: An open access resource for identifying the causes of a wide range of complex diseases of middle and old age. PLoS Med. 2015;12:1‐10. 10.1371/journal.pmed.1001779 PMC4380465 25826379

[23] Bycroft C , Freeman C , Petkova D , et al. The UK biobank resource with deep phenotyping and genomic data. Nature. 2018;562:203‐209. 30305743 10.1038/s41586-018-0579-z PMC6786975

[24] Alfaro‐Almagro F , Jenkinson M , Bangerter NK , et al. Image processing and quality control for the first 10,000 brain imaging datasets from UK biobank. Neuroimage. 2018;166:400‐424. 29079522 10.1016/j.neuroimage.2017.10.034 PMC5770339

[25] Wilkinson T , Schnier C , Bush K , Rannikmäe K , Henshall DE . Identifying dementia outcomes in UK biobank: a validation study of primary care, hospital admissions and mortality data. Eur J Epidemiol. 2019;34:557‐565. 30806901 10.1007/s10654-019-00499-1 PMC6497624

[26] Hemani G , Zheng J , Elsworth B , et al. The MR‐base platform supports systematic causal inference across the human phenome. Elife. 2018;7:e34408. 29846171 10.7554/eLife.34408 PMC5976434

[27] Dale C , Fatemifar G , Palmer TM , et al. Causal associations of adiposity and body fat distribution with coronary heart disease, stroke subtypes and type 2 diabetes: a Mendelian randomization analysis. Circulation. 2017;135:2373‐2388. doi:10.1161/CIRCULATIONAHA.116.026560 28500271 PMC5515354

[28] Bellenguez C , Küçükali F , Jansen IE , et al. New insights into the genetic etiology of Alzheimer's disease and related dementias. Nat Genet. 2022;54:412‐436. 35379992 10.1038/s41588-022-01024-z PMC9005347

[29] Garfield V , Salzmann A , Burgess S , Chaturvedi N . A guide for selection of genetic instruments in Mendelian randomization studies of type 2 diabetes and HbA1c: toward an integrated approach. Diabetes. 2023;72:175‐183. 36669000 10.2337/db22-0110 PMC7614590

[30] Burgess S , Bowden J . Integrating summarized data from multiple genetic variants in Mendelian randomization: bias and coverage properties of inverse‐variance weighted methods. 2015 http://arxiv.org/abs/1512.04486

[31] Bowden J , Davey Smith G , Haycock PC , Burgess S . Consistent estimation in Mendelian randomization with some invalid instruments using a weighted median estimator. Genet Epidemiol. 2016;40:304‐314. 27061298 10.1002/gepi.21965 PMC4849733

[32] Garfield V , Farmaki AE , Fatemifar G , et al. The relationship between glycaemia, cognitive function, structural brain outcomes and dementia: a Mendelian randomisation study in the UK biobank. Diabetes. 2021;23:1140‐1149. doi:10.2337/db20-0895 33632741

[33] Qiao Q . Is the current definition for diabetes relevant to mortality risk from all causes and cardiovascular and noncardiovascular diseases? Diabetes Care. 2003;26:688‐696. 12610023 10.2337/diacare.26.3.688

[34] Yuan S , Mason AM , Burgess S , Larsson SC . Differentiating associations of glycemic traits with atherosclerotic and thrombotic outcomes: Mendelian randomization investigation. Diabetes. 2022;71:2222‐2232. 35499407 10.2337/db21-0905 PMC7613853

[35] De Bresser J , Kuijf HJ , Zaanen K , et al. White matter hyperintensity shape and location feature analysis on brain MRI; proof of principle study in patients with diabetes. Sci Rep. 2018;8:1893. 29382936 10.1038/s41598-018-20084-y PMC5789823

[36] Burgess S , Thompson SG . Avoiding bias from weak instruments in mendelian randomization studies. Int J Epidemiol. 2011;40:755‐764. 21414999 10.1093/ije/dyr036

[37] Fry A , Littlejohns TJ , Sudlow C , et al. Comparison of sociodemographic and health‐related characteristics of UK biobank participants with those of the general population. Am J Epidemiol. 2017;186:1026‐1034. 28641372 10.1093/aje/kwx246 PMC5860371

[38] Lyall DM , Quinn T , Lyall LM , et al. Quantifying bias in psychological and physical health in the uk biobank imaging sub‐sample. Brain Commun. 2022;4:fcac119. 35651593 10.1093/braincomms/fcac119 PMC9150072

